# Informal workers’ access to health care services: findings from a qualitative study in the Kassena-Nankana districts of Northern Ghana

**DOI:** 10.1186/s12914-018-0159-1

**Published:** 2018-05-16

**Authors:** James Akazili, Samuel Chatio, John Ele-Ojo Ataguba, Isaiah Agorinya, Edmund Wedam Kanmiki, Osman Sankoh, Abraham Oduro

**Affiliations:** 1 Navrongo Health Research Centre, P.O Box 114, Navrongo, Ghana; 2 INDEPTH Network, P.O Box KD 213, Kanda, Accra Ghana; 3 Health Economics Unit, University of Cape Town, Cape Town, South Africa; 4 School of Public Health, Faculty of Health Sciences, University of the Witwatersrand, Johannesburg, South Africa; 5 Department of Mathematics and Statistics, Njala University, Njala, Sierra Leone

**Keywords:** Informal workers, Health care services, Northern Ghana

## Abstract

**Background:**

Over the past two decades, employment in the informal sector has grown rapidly in all regions including low and middle-income countries. In the developing countries, between 50 and 75% of workers are employed in the informal sector. In Ghana, more than 80% of the total working population is working in the informal sector. They are largely self-employed persons such as farmers, traders, food processors, artisans, craft-workers among others. The persistent problem in advancing efforts to address health vulnerabilities of informal workers is lack of systematic data. Therefore, this study explored factors affecting informal workers access to health care services in Northern Ghana.

**Method:**

The study used qualitative methodology where focus group discussions and in-depth interviews were conducted. Purposive sampling technique was used to select participants for the interviews. The interviews were transcribed and coded into emergent themes using Nvivo 10 software before thematic content analysis.

**Results:**

Study participants held the view that factors such as poverty, time spent at the health facility seeking for health care, unpleasant attitude of health providers towards clients affected their access to health care services. They perceived that poor organization and operations of the current health system and poor health care services provided under the national health insurance scheme affected access to health care services according to study participants. However, sale of assets, family support, borrowed money from friends and occasional employer support were the copying strategies used by informal workers to finance their health care needs.

**Conclusion:**

Most of the population in Ghana are engaged in informal employment hence their contribution to the economy is very important. Therefore, efforts needed to be made by all stakeholders to address these challenges in order to help improve on access to health care services to all patients particularly the most vulnerable groups in society.

## Background

Over the past two decades, employment in the informal sector has grown rapidly in all regions including low and middle income countries. However, these forms of employment are those jobs that generally lack basic legal protections or employment benefits [1]. About 20% of the world’s working population are actively engaged in the informal sector and contributing significantly to the global Gross Domestic Product (GDP) [2]. In the developing countries, (on average) between 50 and 75% of the people are employed in the informal sector [3, 4].

In Ghana, more than 80% of the working population is engaged in the informal sector where there is lack of social or legal protection [5]. Generally, the sector consists of unregistered private enterprises engaged in the production of goods or services for sale. The larger proportion of the population in Ghana is engaged in the informal sector because of the structural adjustment program that took place in the mid-1980s couple with the inability of the formal sector to generate jobs for the increasing population [5, 6]. The informal workers in Ghana generally comprise farmers, traders, food processors, artisans and craft-workers. Majority of these people are engaged in agricultural activities [5].

Apart from the general lack of labour protection and precarious working conditions, many informal workers may be prone to certain challenges (such as unaffordable out-of-pocket payments, time spent traveling to the health facility and long waiting time before they are attended to by health services providers) in using the needed health services. In some cases, they would have to forgo some wage or the work they are engaged in to enable them use health care services. This adversely affects their welfare including their wellbeing [4, 7].

In order to ensure that all Ghanaians are able to use the needed health care services and to reduce out-of-pocket payment, the government launched the National Health Insurance Scheme (NHIS) in 2004 [8]. Though, the NHIS faces many challenges, it aims to cover the poor and vulnerable groups [9]. In 2008, the scheme included free care for all pregnant women for all maternal health care services [10]. While this is the case, the scheme only makes provision for informal workers to contribute premiums that may not be affordable in order for them to benefit from the NHIS. Quite apart from that these premiums are regressive and the heterogeneity of informal workers is not taken into account [11].

The growing global movement towards universal health coverage (UHC) however provides an opportunity for health systems to address the issue of providing comprehensive health care services for all those who need them, including informal workers at an affordable cost. In order to achieve this, there is a need to understand the barriers that different population groups face in accessing health care services. But there is a dearth of studies examining the specific barriers that informal workers face even though they comprise the bulk of the working population in Ghana and in sub-Saharan Africa. In some cases, these workers are considered among the poor and assumed to face the same sets of barriers [12, 13]. Although, some of the problems poor people may face in accessing health care services may be similar to those faced by informal workers, the latter group encounters specific barriers related to the nature and context of their unstable employment and unfair working conditions [14, 15]. In this regard and in the context of UHC in Ghana; this study explored factors affecting informal workers’ access to health care services in the Kassena-Nankana Districts of Northern Ghana.

## Methods

### Study design

The study was exploratory using qualitative approach where Focus Group Discussions (FGDs) and In-Depth Interviews (IDIs) techniques were used to gather data. The study population was informal workers engaged in subsistence farming, hairdressing, casual work, beer bar attendants and female head-porters popularly known as “kayaye” in Ghana. Hairdressing, for instance, is a fast growing business that absorbs a large proportion of young ladies who are self-employed and with minimal education. Kayaye is a form of employment for unskilled and uneducated internal migrants mostly women from the northern part of Ghana in search of jobs [16]. They usually carry goods and wares on the head for shoppers and traders in and around commercial centres for a petty fee. The kayaye have no regulated market and they face precarious working conditions that may have negative effect on their health. For instance, lifting heavy loads and travelling long distances with the loads. Subsistence farmers comprise a major group especially in rural areas where the bulk of the Ghanaian population resides and this thrives on the ancestral land ownership system. Causal workers are usually informal workers employed in the formal sector. This is a growing phenomenon in Ghana as these workers are hired for a short period of time without any signed contract and the nature of their work, including the uncertainties with which they work create several challenges that impact negatively on their health. Beer bar employment is also another growing area in the study area where young people are engaged by bar owners to provide services to customers for a petty monthly fee. These are mostly young girls who have completed their Senior High School education and for them to make a living, they are engaged casually to serve in these bars.

The FGDs and the IDIs with subsistent farmers, casual workers and the hairdressers were conducted in the Kassena-Nankana East and West Districts (KNEWDs) while the FGDs with “kayaye” were conducted in the urban area, Accra Metropolitan in Southern Ghana. This was done because kayaye are not found in rural areas in Ghana. Study participants shared their views on factors affecting their access to health care services and suggested ways to improve access to health care services.

### Study site

The primary site for this study is rural Kassena-Nankana East and West Districts of Northern Ghana. The districts cover an area of 1675 km^2^ of Sahelian savannah with a population of about 153,000 people [17]. The main languages spoken in the area are Kasem and Nankani. The population is predominantly rural with subsistence farming as the mainstay of the districts’ economy. People live in multi-household compounds in these districts. There are two distinct seasons; the raining season which spans from May to October with the rest of the year being dry. The main health problem in the area is malaria with the highest transmission period occurring between June to October [18, 19].

The districts have one hospital, eight health centers, two private clinics, which provide curative and preventive health care services to community members. There are about 28 Community-based Health Planning and Services (CHPS) compounds located in various communities and providing reproductive health services and treatment of minor health conditions [20]. The district hospital located in Navrongo Township serves as a referral point for all health facilities operating in the two districts.

### Training of data collectors

Two university graduates with experience in conducting qualitative interviews were recruited and trained for data collection. The training covered areas such as the purpose of the study and qualitative interviewing techniques. Role plays were done during the training session to help data collectors understand the interview guides. A pre-test was also conducted at the end of the training. The essence of the pre-test was to help evaluate the performance of data collectors and also help the study team finalize the interview guides before the actual data collection.

### Sampling procedure/ data collection technique

The study population involved all informal and causal workers in the study area. Therefore, all male and female informal workers in the study area qualified to be part of the study. Purposive sampling technique was used to select study participants for the interviews. Study participants were identified and invited for the interviews. For the FGDs, suitable arrangements such as date, venue and time were made prior to the conduct of the interviews at the community level. Appointments were also booked with the IDI participants before the interviews were conducted at home. All the interviews were tape recorded with consent from participants. The interviews in the KNDs were conducted in the main local languages spoken in the area (Kassem and Nankani) while twi which is predominantly spoken in Southern Ghana was used to conduct the interviews with the kayaye. A total of 6 FGDs (2 with subsistent farmers, 2 with kayaye, 2 with casual workers) and 15 IDIs (5 with hairdressers, 5 with beer bar attendants, 5 with subsistent farmers) were conducted. The FGDs were made up of a minimum of 8 to a maximum of 10 participants per group.

### Data processing and analysis

We utilized the principle of saturation for data collection in this study. Data saturation was reached where no new or additional information is being found from the interviews [21]. Data collection, management and analysis were done concurrently. This was done to ensure that new themes were incorporated into the guides and to also ensure that data were collected to cover all the thematic areas. All interviews were audio-recorded and later transcribed verbatim after repeatedly listening to the recordings. The transcripts were then uploaded onto QSR Nvivo 10 software to facilitate data management and coding [22]. The transcripts were coded by two independent researchers and this was done to ensure a fair interpretation of the data. Guided by the objectives of the study, the coding process involved a critical review of each transcript to identify emerging themes from the data. The two coders then met to compare their independently-identified themes. They revolved divergence by re-reading the relevant sections of the transcripts together, and agreed on the best fit interpretation of the data. The major and sub-themes are discussed below, supported by relevant quotes from the transcripts.

### Ethical considerations

The protocol was approved by the Navrongo Health Research Centre Institutional Review Board before the commencement of fieldwork. Verbal consent was obtained from study participants before the interviews were conducted. Verbal consent was obtained because the study processes posed minimal risk to study participants. In addition, this method of consent was solicited and obtained as the majority of the respondents had no formal education. The purpose of the study, potential benefit and the right to withdraw from the study were explained to study participants before the interviews were conducted. To ensure confidentiality and anonymity, codes were assigned to study participants instead of their names.

## Results

### Background information of participants

In all, 68 participants were interviewed in this study. The FGD participants were 53 while 15 people took part in the IDIs. Thirty-nine participants were males while twenty-nine of them were females. The ages of the participants were grouped into three categories (21–30 years, 31–40 years and 41–50 years). Most of them (twenty nine) were between 31 and 40 years old. Twenty-seven were 21–30 years while only twelve of them fell between 41 and 50 years old. Majority (twenty-four) of the participants completed at least senior high level education, sixteen went up to junior high while eleven of them had primary education. However, seventeen of them never went to school. In terms of religion, majority (forty-three) were Christians, sixteen were Muslims while only nine of them worshiped the traditional religion.

### Factors affecting access to health care services

Several health system and individual level factors were mentioned by study participants that affected their access to health care services. These factors are discussed below:

### Time spent at the health facility for medical care

Study participants reported that they spent a lot of time at the health facility seeking for medical care and that really affected their daily productivity. According to participants, they could have used that time productively at their work places. For them, this greatly discouraged their visit to the health facility for health care services. As a 32 year old hairdresser put it:

*“In fact that one is a problem because you may be having a lot of work and when you visit the health facility, you will spend more time there trying to get treatment. You have to follow the line for long before you get to the doctor and because of that sometimes you even say that when you go to the drugstore and spend money buying your drugs quickly and come back and do your work, it is better”* **(32 year old hairdresser-IDI).**

For the causal workers, one could even lose his/her work for consistently visiting the health facility for medical care. In one of the FGDs with the causal workers, a participant had this to say on the issues:

*“You see if you are a contract worker you may not have the time to go and queue in order to get health service… and that is the reason why we the contract workers are at a disadvantage. For us ( the contract workers) when they know that you are somebody who easily falls sick and goes to the hospital instead working, they may sack you or terminate your contract. For that reason sometimes you may be sick and yet you will be compelled to go to work because you are on contract and they will not tolerate you when you are always asking for permission to go to hospital*” **(Casual worker-FGD)**

### Unavailability of medicine and health personnel

Unavailability of medicines at the health facilities and poor medical services were also reported by study participants. Most of them said that unavailability of medicines for them at the health facility affected their access to these medicines. They reported that patients were given prescriptions to buy their medicines at the drugstore after spending the whole day at the health facility. For them, they saw it necessary to visit the drugstore right away and buy their medicines instead of going to the hospital to spend several hours collecting only prescriptions. Unavailability of health personnel to attend to patients at the health facility was also reported by few of the study participants. In their own words, discussants presented their views this way on the issue:

*“My child was sick and I took the child to the hospital and when we went to the hospital instead of them giving us medication, they rather gave us prescription for us to buy the drugs at the chemical shop. So it has made me think that when you are sick it is better to go and buy your drugs and use instead of going to spend time at the hospital means while you will not even get medicine”* **(33 year old hairdresser-IDI).**

*“When you go to the health facilities, even common eye drop they don’t have it and sometimes you wonder why it is so. Even laboratory test they don’t do it at the hospital, they rather refer you to the private lab just closer to the hospital. I recently took my sick child to the hospital and when we got there and after the folder was given to us, there was no doctor to attend to us and at the end of the day we came back home like that without anybody attending to the child. We came back and use local treatment*” **(40 year old subsistent farmer-IDI)**

### Perceptions on medicines given to patients at the health facility

The quality of medicines was also reported as not the best by study participants. They perceived that the medicines given them at the health facility were not of good quality. They added that the health insurance which was introduced to benefit the poor was rather a problem for them in terms of their access to good medicines especially at the public health facilities. They said this was not the best because it encouraged them to buy medicines at the drugstores instead of visiting the health facility for medical care.

*“Oooh, for the health insurance, it has a lot of disturbances because if not bed and paracetamole what again do they give? They are the only things they do for us at the hospital under the health insurance. When you visit the health facility with your health insurance card, you will not get good drugs”* **(30 year old hairdresser-IDI).**

*“A farmer is a poor person because it is not all the time you will have good harvest. When you manage to register with the health insurance and you fall sick and visit the hospital, it is only paracetamole they will give. They don’t have good medicine for you at the hospital to help you recover well and go back and continue with your work. So I must say that the medicine they give us at the hospital is really not good and for me, all that is because of the health insurance that is not helping us”* **(Subsistent farmer-FGD).**

### Health system drawbacks

Poor operations of the current health system as a result of the implementation of the National Health Insurance Scheme also placed a very big challenge on access to health care services according to study participants. The inability for the National Health Insurance Scheme authorities to reimburse money to health facilities and accredited chemical shops was reported by study participants as one barrier affecting their access to health care services. They held the view that this had made the accredited chemical shop operators refused to give them medicines anytime they went there with prescriptions. They also perceived that the little money that was given to the health facilities, the authorities were not using the money to buy the needed medicines and other equipment to enable them provide health care services to patients.

*“The implementation of the health insurance is also a problem. The reason is that when you go to the health facilities, they say that the government does not pay (reimburse) them the money and that is why they are unable to give us the drugs. So the services are poor there”* **(Casual worker-FGD)**

*“What I have to say is that sometimes the government will give the money but the authorities at the health facilities are feeling lazy to purchase the drugs if not why is it that the drugs are at the chemical shop and they are not at the health facilities? When they give them the money they will take 50% of it into their pockets and use just small amount to buy some few drugs and they will now come and say government does not pay them and things like that”* **(22 year old beer bar attendant-IDI)**

### Negative attitude of health workers towards clients

Study participants also reported unprofessional conduct of some health workers as not the best. They said it was another factor that deterred them from visiting the health facilities for health care services. Health workers were reported to be verbally abusive and because patients did not find it pleasant to have such encounters with health workers, discouraged them from seeking health care services at the health facilities. In their own words, respondents gave examples on the negative conduct of health workers towards their clients especially at the public health facilities:

*“The health workers attitude at the public health facilities is not good and sometimes you ask yourself this question why they behave that way towards sick people…”* **(Casual worker-FGD)**

*“For the health worker’s, their behavior is not good at all because I remember the time I went there to deliver they did something to me such that I will never forget. I was in labour and when I went, the doctor was not present. Is like it was not time for me to deliver and once the time is not due for you to deliver they (nurses) will not mind you just because the time is not up for you to deliver but the sufferings that you are going through, they will not mind you at all”* **(Kayayo-FGD)**

### Affordability

The inability to register with the health insurance scheme or pay for the cost of medical services at the health facilities as a result of lack of money was also reported by participants as one of the barriers affecting access to health care services. The informal workers attributed this to poverty or low earnings mainly because of the kind of work they were engaged in. Participants shared their views this way on their inability to afford health care services:

*“The problem is the poverty; we do not have the money to go and pay for drugs at the hospital or to register with the health insurance. We don’t get anything in this work that we do. You can get up the whole day and you will not get somebody to carry his/her things in order to get money and even buy food and eat….”* **(Kayayo-FGD).**

*“The issue has to do with the money because when you fall sick and you are not registered with the insurance, you will not have money to go and buy folder and the medicine at the hospital because you don’t even have it to buy food. The little money you have, you can just go and buy drugs at the drugstore and take to see whether your condition it will be better”* **(20 year old beer bar attendant-IDI)**

### Coping strategies for health care and health care financing

Most of the participants reported that they used the little money available to buy medicine anytime they fell ill. They added that they sometimes borrowed money from friends and relatives to take care of their medical bills. Participants however mentioned that it was not all the time they could get support from relatives and friends. Some of them especially the farmers sold assets or their farm products to buy medicine at the drugstore anytime they fell sick.

*“It is still my mother who gives me money to buy medicine anything I fall sick because this work that we do, the money they pay us is small. It is very small for you to use it and buy your needs and still save some of it to buy medicine when you are sick. It is my mother who usually gives me money to go to the hospital whenever I am sick”* **(21 year old bar attendant-IDI)**

*“When you are not registered with the insurance and you are sick and you don’t have money to pay for the medical bills, you have to sell one animal before you can get money to pay for the medical bill. So these are some of the problems that we face because you will end up selling all your farm produce to take care of your health needs”* **(Subsistent farmer-FGD).**

Employer support to access health care services was mentioned in the discussions. Discussants said that employees including casual workers who had very serious health problems and the services were not covered by the national health insurance scheme were supported by the organization. This kind of support was only mentioned by the casual workers in the group discussions. A participant in one of the group discussions with casual workers offered his views this way on the issue:

“*You see the organization was supporting us to take care of our health needs and when the health insurance system was introduced, they had to stop the free health care services that we were enjoying. What is happening now is that they only pay for very expensive drugs that the health insurance does not cover or when you have very serious health issues for instance when you have a broken leg and they refer you, the organization will support you financially*” **(Casual worker-FGD).**

Few participants especially the subsistent farmers reported that they sometimes used local medicine to treat their condition. They said that it was because of their inability to register or renew their health insurance cards made them to use herbal medicine whenever they fell sick and they did not have money to visit the health facility for modern health care services.

*“When you don’t have money to go to the hospital, you have to use the local treatment like my colleagues mentioned earlier. Sometimes when you boil the neen leafs (particular tree use by community members to treat malaria) and drink and also bath with the water, you may feel better. It will not cure you completely but it will be better for you to go on with your work*” **(45 year old subsistent farmer-IDI).**

### Suggested ways to improve access to health care services

Study participants proposed various strategies to help improve access to health care services. They stated that the restructuring of the national health insurance policy by the government could help solved the current challenges in the health system. Participants were of the view that prompt reimbursement of money to the facilities by the health insurance authorities could help them to provide the needed health care services to clients. They suggested the need for effective supervision at the health facility level to make sure that prescriptions given to patients to buy drugs at the drugstores were actually drugs that were either not available at the facility or they were not covered by the health insurance.

*“For me, the health insurance people should have a policy such that when prescriptions are given to patients to buy drugs, they should cross check and see whether it is covered by the insurance or not and if it is covered why is it that the health facility does not have it! When they do that I think it will help solve some of these problems where you will visit the health facility and you will not get drugs. The government should also try and pay (reimburse) all the monies to the facilities to enable them buy all the necessary drugs and give to patients”* **(Subsistent farmer-FGD)**

With regards to the unpleasant behavior of some of the health workers, most participants were of the view that it was important for health workers to have patience and behave nicely towards clients in a manner that would encourage them to always feel free to visit the health facility for health care. A participant in one of the group discussions had this to say on the issue:

*“Most of the nurses do not have good behavior at all and that is also contributing to poor health care services provided to clients at the public health facilities. They should be good human relation between the health workers and the patients they provide the services to. So I think it is the discipline that they lack and so if discipline is introduced at the health facilities, they will do the right thing”* **(Casual worker-FGD)**

The subsistent farmers also suggested that the government could reduce the registration fee to enable people in their category to register and also renew their health insurance cards anytime they expired. They added that the renewal period could be extended to five years instead of the yearly renewal which they could easily forget.

*“…I suggest that they should allow us (farmers) to register with the health insurance for five year period for the health insurance card instead of the yearly registration and renewal. If you pay at once for five years, that will be better. The government can also reduce the registration fee to enable many of us to register”* **(Subsistent farmer-FGD)**

## Discussion

Good health is very important for all workers especially those working in the informal sector because good health leads to increase in productivity [23]. Based on this, it is very important for informal workers to have access to health care services any time they need these services. The NHIS currently operating in Ghana requires that people are supposed to pay and register with the health insurance scheme to enable them receive free health care services anytime they fall sick [8]. However, various factors in this study have been reported as responsible for the inability of informal workers to register with the insurance scheme to enable them have access to health care. This has been attributed to the low income earned by informal workers. This is consistent with previous study that reported that vulnerable groups had lower access to health care services because of poverty [12]. Most of the informal workers are petty traders and some are engaged in jobs that they do not earn enough income to be able to save and take care of their health needs. It is therefore not surprising that most of the participants in this study reported their inability to register with the national health insurance or finance their health care needs because of the low income that they earn.

There are usually long queues at the Out Patient Department (OPD) level because of the large number of patients who visit the health facilities daily for health care services. Informal workers are self-employed and because of that they need time to carry out their daily activities. As a result, they prefer not to go and spend much time at the hospital/health facility if the condition is not very serious. Participants held the view that this has contributed largely to affect access to health care services by informal workers. The reason is that most of them are engaged in their own private work and would not want to spend the whole day at the health facility seeking for medical care with the neglect of their work. Based on this assertion, informal workers in the study area considered their health less important than the work they are engaged in. It is demonstrated that negative attitude of health professional towards clients affected access to health care services [12]. Where patients are not being treated very well by health professional, it could prevent them from visiting the health facility for health care services. Informal workers are vulnerable group and because of that health workers could treat them unfairly at the health facility. This therefore does not encourage them to visit the health facility for health care when necessary. This leaves them no option than to buy medicine at the drugstore or use herbal medicine to treat their condition as mentioned in this study by informal workers.

It is revealed that distance to service point, lack of skills staff at public health facilities and lack of knowledge affected access to health care services [12, 13]. The same studies also reported cultural beliefs and practices leading to self-care at home and consultation with traditional healers in rural communities discouraged people from seeking for health care services at the health facilities [12, 13]. These factors however have not been reported in this study. Though, some of them especially the subsistent farmers mention that they sometimes use local medicine (herbs) to treat their illnesses, it is not a barrier that prevents them from visiting the health facility for medical care. The issue had to do with their inability to pay for health services at the health facilities left them with no option than to use these herbs.

Poor organization, planning and operations of the current health system as a result of the introduction of the national health insurance policy according to study participants seriously affected health care services especially low income earners and the poor. The health insurance authorities’ are not able to reimburse money to the accredited chemical shop operators and health facilities to enable them provide quality health services to clients. This therefore leads to the issue of poor health care services and unavailability of medicines at the health facilities. It is demonstrated that health system challenges and poor health care services provided had negative influence on access and utilization of health care services [24, 25].

Most of the working population in Ghana is found in the informal sector and majority of them are not able to have access to health care services as a result of the factors discussed earlier. Study participants in this study however suggested various ways that could help address these issues in order to enable them have access to health care. Effective supervision and the commitment by the health insurance authority to reimburse the money they own to health facilities could help address the issue of unavailability of medicines especially at the public health facilities according to study participants. The registration fee for the NHIS could also be reduced to enable the poor or low income workers such as informal workers to register and also renew their health insurance cards anytime they expire. Though tax revenue is one of the main sources of health care financing by most governments, health promotion campaign is needed through inter-sectorial collaboration focusing on the more disadvantage population on the need for them to save towards their health needs [26].

### Limitations

Though this study offers requisite information on factors affecting informal workers access to health care services in the study area, our study has some limitations. This was a qualitative study using a non-probability (purposive) sampling method to select study participants to share their personal views and experiences on the issue which may not necessarily represent the views of the larger population. Therefore the finding of this study could not be generalized to the larger population in Ghana. It is however recommended that similar studies could be carried out in other parts of the country in order to obtain holistic views about the issue for a national decision to be taken to address these concerns. Also, the interviews were mostly conducted in the main local languages and translated into English. Therefore some words could have lost their original meaning. To help minimize this, two independent people were made to do the translations and transcriptions which were verified. However, given the limitation of such a method, in doing the analysis emphasis was placed on the predominant themes and not specific word choices or phrases. Also, information on participants’ health insurance status was not collected and this did not allow us to analyze and report on the number of study participants’ registered with the NHIS and the number of those who did not register.

## Conclusion

Most of the population in Ghana is engaged in informal employment and their contribution to the economy is very important. However, there are various factors that affect their access to health care services especially at the public health facilities. Factors such as poverty, the unpleasant attitude of some health workers towards clients and poor health system organization and operations as a result of the introduction of NHIS in Ghana negatively affect access to health care services of informal workers. It is therefore, recommended that much efforts needed to be adopted by the government, Ghana health service, Ministry of health, the health insurance authority and other stakeholders to address these challenges. This will help improve on the general health care delivery not only for the informal workers but also to the poor and most vulnerable population in Ghana who are mostly in need of these services.

## Abbreviations

CHPS: Community-Based Health Planning and Services
FGD: Focus Group Discussion
GDP: Gross Domestic Product
IDI: In-Depth Interview
KNEWDs: Kassena Nankana East and West Districts
OPD: Outpatient Department
UHC: Universal Health Coverage
